# Synthesis and a Photo-Stability Study of Organic Dyes for Electro-Fluidic Display

**DOI:** 10.3390/mi11010081

**Published:** 2020-01-11

**Authors:** Yong Deng, Shi Li, Dechao Ye, Hongwei Jiang, Biao Tang, Guofu Zhou

**Affiliations:** 1 Guangdong Provincial Key Laboratory of Optical Information Materials and Technology & Institute of Electronic Paper Displays, South China Academy of Advanced Optoelectronics, South China Normal University, Guangzhou 510006, China; yong.deng@guohua-oet.com (Y.D.); shili@m.scnu.edu.cn (S.L.); hongwei.jiang0822@gmail.com (H.J.); guofu.zhou@m.scnu.edu.cn (G.Z.); 2 Shenzhen Guohua Optoelectronics Tech. Co. Ltd., Shenzhen 518110, China; dechao.ye@guohua-oet.com; 3 Academy of Shenzhen Guohua Optoelectronics, Shenzhen 518110, China

**Keywords:** electro-fluidic display, organic dye, colored oil, photo-stability

## Abstract

Electro-fluidic display (EFD) is one of the most promising reflective displays because of its full color and video speed. Colored EFD oil, which normally consists of soluble organic dyes and non-polar solvent, plays a critical role in color, electro-optical behavior, and the reliability of the EFD devices. In this paper, we report our research on two kinds of electro-fluidic dyes based on anthraquinone and azo pyrazolone, including their synthesis, structure characterization, and application properties. Changes of absorbance curves, color coordinates of oils, and photoelectric responses of devices were studied in detail under accelerated irradiation to investigate the photo-stability and reliability properties of synthesized oil materials and devices. Photoelectric responses and photo stability of dyes are highly varied depending on their structures. We found that 1,4-dlialkylamino anthraqinone and mono azo pyrazolone dyes are much more stable than 1,8-dlialkylamino anthraqinone and corresponding bisazo pyrazolone dyes.

## 1. Introduction

Recently, electro-fluidic display as an emerging display technology has received widespread attention [1,2,3,4]. It has many advantages: (1) a reflective mode for using ambient light and energy saving [5]; (2) quick response (its switching time is less than 20 ms) for video display [6]; (3) superb optical performance (its white state reflectance is up to 50% [7] and it has full color) [8]; and (4) fluidic and soft display candidate materials for flexible displays.

Colored oils act as optical switches in electro-fluidic display, which affect not only the color gamut but also the contrast ratio, electro-optical response, and the lifetime for outdoor display devices. High optical density, good solubility in non-polar solvent, and good light stability are the main characteristics for the designation of soluble organic dyes. A series of efforts to develop efficient dyes for electro-fluidic display have been conducted in previous research, such as the designation of anthraquinone dyes [9,10], azo dyes [11,12,13,14,15], dipyrrole methane metal dyes [16], and pigment dispersion [17,18]. However, for many of the dyes that were disclosed in patents, few were subjected to detailed research on their synthesis and specific application properties, especially their photo-stability properties.

In our opinion, the photo-stability of the oil materials is one of color dyes’ most crucial properties, which could limit their industrialization. In their designation lifetime, color dyes will decompose and their color image as well as their response properties will deteriorate. In this context, we have synthesized two typical electro-fluidic display (EFD) dyes (anthraquinone and azo pyrazolone dyes), and their electro-optical behavior, photo-stability properties, and structure correlation were researched in detail in this paper.

## 2. Experimental

### 2.1. Materials

All of the color dyes were synthesized in our laboratory. The structures were characterized and proved by using several analysis methods: Infrared (IR) (PerkinElmer, Shelton, CT, USA), Nuclear magnetic resonance (^1^H NMR) (Bruker BioSpin GmbH, Rheinstetten, Germany), ^13^C NMR (Bruker BioSpin GmbH, Rheinstetten, Germany), High resolution mass spectra (HRMS/ESI) (Bruker Daltonics, Bremen, Germany)). Indium Tin Oxide (ITO) coated glass (0.7 mm thickness) with a resistance of 100 Ω·m (purchased from Shenzhen Laibao Hi-Tech Co. Ltd., Shenzhen, China) was used as the bottom and top substrates. SU-8 3005 photoresist was purchased from Microchem Corp. (Newton, MA, USA). AF Teflon^®^ 1600X was purchased from Dupont^TM^ Co. (Wilmington, DE, USA)

### 2.2. Analysis Methods

UV-3300 spectrophotometer (Jinpeng Analytical Instrument Corporation, Shanghai, China) was used to record UV-vis (ultraviolet) absorption spectra of synthesized dyes. Perkin–Elmer 841 spectrometer (PerkinElmer, Shelton, CT, USA) using KBr pellets was used to record FTIR spectra. Varian AS400 (Agilent, Santa Clara, CA, USA), Bruker AVANCEIII 500 (Bruker BioSpin GmbH, Rheinstetten, Germany) and AVANCE NEO Bruker 600 (Bruker BioSpin GmbH) were used to record ^1^H NMR and ^13^C NMR spectra in deuterated solvents using tetramethylsilane (TMS) as an internal standard. Bruker Daltonics Microflex mass spectrometer (Bruker Daltonics, Bremen, Germany) was used to record the mass spectra. The thickness of the AF layer and height of the pixel wall were measured by using the Dektak (Dektak XT, BRUKER, Hamburg, Germany). A self-assembling oil filling instrument was made by our team. The photo-stability of Four dyes in EFD cell was tested in a Xenon arc lamp weather resistance test chamber (B-SUN-I), purchased from Shanghai Yiheng Instruments Co. Ltd (Shanghai, China). The simulation conditions were set according to international standard IEC 60068-2-5: Procedure B; 0.55 W/m^2^ (340 nm); Temperature 45 °C. Absorbance curves before and after a period of irradiation were tested.

### 2.3. EFD Oil Formulation

Certain quantities of dyes and decane were weighted in a sample cell and put in the ultrasonic instrument for 30 min. Afterwards, the solution was subjected to a filtration process using a 0.45 μm filtrator to remove suspended impurities. Then the well prepared oil was used in the next EFD device fabrication process.

### 2.4. Electro-Fluidic Display (EFD) Device Fabrication

The EFD device was fabricated as follows: a 8 × 6 cm^2^ ITO coated glass was used as a bottom substrate. A Teflon (AF Teflon^®^ 1600X, Dupont^TM^ Co.) layer of 0.8 μm was spin-coated on top of the ITO glass as the dielectric layer and hydrophobic layer. A pixel wall structure was fabricated by standard lithography process using photoresist material (SU-8 3005). After coating a photoresist film on active fluoropolymer surface, an exposure process was used to obtain a patterned reacted resist layer. Finally, a 7.5 μm height pixel wall with a 15 μm width was developed. The square of each pixel was 150 × 150 μm^2^. After that, oil with an average thickness of 4.5 μm was filled into pixels by a self-assembly machine. The last step of the fabrication processes was to integrate another top ITO glass with a gap of 75 μm filled by a water phase.

## 3. Dye Synthesis and Characterization

**Figure 1:**
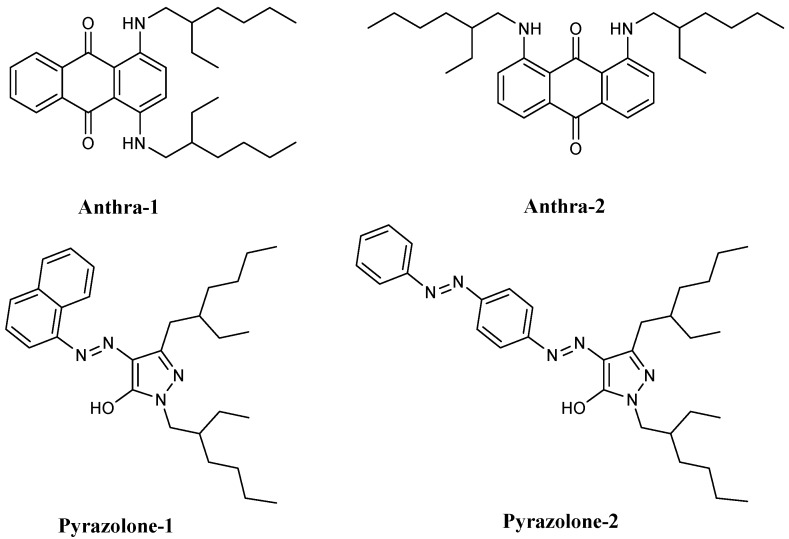
Molecular structures of the four synthesized dyes.

The structure of four designed dyes were shown in the Figure 1.

Anthra-1: 2.0 g (242 g/mol, 0.0082 mol) Anthracene-1,4,9,10-tetraol was dissolved in 20 mL 2-methoxyethanol in a three-necked flask with agitation, and the solution was heated to 80 °C under an N_2_ atomosphere. Then, 4.26 g (129 g/mol, 0.033 mol) 2-ethyl-hexylamine was added once. The solution was refluxed for 24 h. After that, the reaction was exposed to air for 2 h at 50 °C. Then the reaction was cooled to room temperature, poured into 100 mL water, and filtrated to get 2.77 g crude products Anthra-1. The crude Anthra-1 was purified by flash chromatography on silica gel (eluting agent: petroleum ether and ethyl acetate). Characterization: cyan solid; yield: 73.1%; *λ* (max) = 650 nm (in decane); *ε* = 17930 L·mol^−1^·cm^−1^. IR (KBr, cm^−1^): 3061.80 (Ar–H); 2962.50 (–CH_3_), 2921.20 (–CH_2_^−^), 2859.10 (–CH_3_); 1645.9 (–C=O); 1581.80 (Ar); 1555.0 (Ar); 1521.90 (Ar); 1459.90 (–CH–); 1379.27(–CH_3_); 1257.28 (C–N–C); 816.89. ^1^H NMR (500 MHz, CDCl_3_): 9.71 (s, 1H, Ar–NHCH_2_^−^), 8.25 (m, 2H, Ar–H), 7.65 (m, 2H, Ar–H), 7.35 (d, 2H, *J* = 19.0, Ar–H), 3.22 (m, 4H, –NCH_2_^−^), 1.73 (m, 2H, –CH–), 1.50 (m, 4H, –CH_2_^−^), 1.26 (m, 6H, –CH_2_^−^),1.18 (m, 6H, –CH_2_^−^), 0.88 (m, 12H, –CH_3_). ^13^C NMR (500 MHz, CDCl_3_): 182.11, 146.56, 134.57, 131.84, 126.03, 123.66, 109.67, 45.99, 39.47, 31.28, 29.04, 24.53, 23.03, 14.12, 10.99. MS (MALDI-TOF) (DIF) M/Z (%): 463.20 (M + H)^+^. Calc. for (C_30_H_42_N_2_O_2_): 462.30.

Anthra-2: 2.0 g (227 g/mol, 0.0088 mol) 1,8-dichloroquinone was dissolved in 20 mL 2-ethyl-hexylamine in a three-necked flask with agitation, and the solution was heated to 150 °C under an N_2_ atomosphere for 12 h. After reaction, the solution was poured in 10% hydrochloric acid solution filtrate to remove excess 2-ethyl-hexylamine and to obtain 1.60 g of crude products Anthra-2. The crude Anthra-2 was purified by flash chromatography on silica gel (eluting agent: petroleum ether and ethyl acetate). Characterization: magenta solid; yield: 40%; *λ* (max) = 545 nm (in decane); *ε* = 13335 L·mol^−1^·cm^−1^. IR (KBr, cm^−1^): 3268.50 (–NH–); 3082.40 (Ar–H); 2962.50 (–CH_3_); 2925.30 (–CH_2_^−^); 2863.30 (–CH_3_); 1658.30 (–C=O); 1623.20 (–C–N–); 1569.40 (Ar); 1531.60 (Ar); 1459.90 (–CH–); 1397.80 (–CH_3_); 1296.50 (C–N–C); 1211.80 (C–N–C); 1073.20; 1019.51; 837.57; 744.53. ^1^H NMR (500 MHz, CDCl_3_): 9.67 (s, 1H, Ar–NH–); 7.45–7.43 (d, 2H, *J* =1 Hz, Ar–H); 7.40–7.37 (t, 2H, *J* = 15.5 Hz, Ar–H); 6.95–6.93 (d, 2H, *J* = 1 Hz, Ar–H); 3.16–3.14 (s, 4H, *J* = 11 Hz, Ar–NH–), 1.65 (m, 2H, –CH–); 1.44 (m, 8H, –CH2–); 1.28 (m, 8H, –CH2–); 0.91-0.88 (t, 6H, *J* =15 Hz, –CH_3_); 0.86–0.83 (t, 6H, *J* = 15 Hz, –CH_3_). ^13^C NMR (500 MHz, CDCl_3_): 188.99, 184.84, 151.48, 134.22, 117.63, 114.63, 46.08, 38.97, 31.49, 28.99, 24.79, 23.06, 14.11, 11.13. MS (MALDI-TOF) (DIF) M/Z (%): 463.20 (M + H)^+^. Calc. for (C_30_H_42_N_2_O_2_): 462.30.

Pyrazolone-1: A quantity of 1.43 g (143 g/mol, 0.01 mol) naphthyl amine was dissolved in 20 mL of ethanol, 10 mL of water, and 3 mL (0.036 mol) of concentrated hydrochloric acid. After it had been rapidly cooled to 0–5 °C, a 5 mL solution containing 0.72 g (69 g/mol, 0.0105 mol) of sodium nitrite was added. Ehrlich reagent was used to detect the termination of diazotisation, and sulfamic acid was used to remove the residual nitrous acid. Afterwards, 2.94 g of the coupling component 1-(2-ethylhexyl)-3-(2-ethylhexyl)-5-pyrazalone (294 g/mol, 0.01 mol) was dissolved in 100 mL ethanol and cooled to 0–10 °C. Diazonium salt solution was added to the above solution at a temperature below 10 °C over a 15–20 min period, with the pH level maintained at 8–9 using a sodium hydroxyl solution. The reaction mixture was stirred for a further 3 h. Pyrazolone-1 was precipitated by adjusting the pH of the coupling solution to 4 using acetic acid, then collected and dried to get 3.5 g of crude product Pyrazolone-1. The crude Pyrazolone-1 was purified by flash chromatography on silica gel (eluting agent: ethyl acetate / petroleum ether = 1/100, v/v). Characterization: yellow solid; yield: 80.5%; *λ* (max) = 420 nm (in decane); *ε* =16119 L·mol^−1^·cm^−1^. IR (KBr, cm^−1^): 3058.90 (Ar–H); 2958.60 (–CH_3_); 2929.70 (–CH_2_^−^); 2856.40 (–CH_3_); 1656.70 (C=O), 1560.40 (Ar); 1521.70 (Ar), 1456.10 (–N=N–); 1392.50 (–CH_3_); 1244.00 (C–N–C); 1091.60, 792.70. ^1^H NMR (500 MHz, CDCl_3_): 14.478 (s, 1H, O–H); 8.00–7.99 (d, 1H, Ar–NH–N=Ar); 7.81–7.76 (d, 2H, Ar–H); 7.61–7.59 (d, 1H, Ar–H); 7.52-7.49 (t, 1H, Ar–H); 7.46–7.43 (m, 2H, Ar–H); 3.65–3.63 (d, 2H, –NCH_2_^−^); 2.66 (m, 1H,–CH–); 1.80 (m, 5H, –CH_2_^−^); 1.24 (m, 12H, –CH_2_^−^); 0.81–0.78 (m, 12H, –CH_3_). ^13^C NMR (500 MHz, CDCl_3_): 159.03, 149.97, 136.36, 134.07, 129.88, 128.75, 127.00-123.52, 121.34, 110.76, 47.84, 38.47, 32.95, 30.65, 28.76, 26.22, 25.45-22.28, 14.11, 10.74. MS (MALDI-TOF) (DIF) M/Z (%): 461.30 (M–H)^−^. Calc. for (C_29_H_42_N_4_O): 462.30.

Pyrazolone-2: A quantity of 1.97 g (197 g/mol, 0.01 mol) 4-aminodiazobenzene was dissolved in 30 mL of water and 3 mL (0.036 mol) of concentrated hydrochloric acid. After it had been rapidly cooled to 0–5 °C, a 5 mL solution containing 0.72 g (69 g/mol, 0.0105 mol) of sodium nitrite was added. Ehrlich reagent was used to detect the termination of diazotisation, and sulfamic acid was used to remove the residual nitrous acid. The coupling component 1-(2-ethylhexyl)-3-(2-ethylhexyl)-5-pyrazalone 3.08 g (308 g/mol, 0.01 mol) was dissolved in 100 mL ethanol and cooled to 0–10 °C. Diazonium salt solution was added to the above solution at a temperature below 10 °C over a 15–20 min period, with the pH level maintained at 8–9 using a sodium hydroxyl solution. The reaction mixture was stirred for a further 3 h. Pyrazolone-1 was precipitated by adjusting the pH of the coupling solution to 4.0 using acetic acid, then collected and dried to get 4.5 g crude product Pyrazolone-2. The crude Pyrazolone-2 was purified by flash chromatography on silica gel (eluting agent: petroleum ether and ethyl acetate). Characterization: yellow solid; yield: 87.8%; *λ* (max) = 416 nm (in decane); *ε* = 25335 L·mol^−1^·cm^−1^. IR (KBr, cm^−1^): 3103.20 (Ar–H); 2958.40 (–CH_3_); 2929.40 (–CH_2_^−^); 2863.30 (–CH_3_); 1695.60 (C=O), 1617.00 (Ar); 1579.80 (Ar); 1521.90 (Ar); 1461.90 (–N=N–); 1379.20 (–CH_3_); 1226.20 (C–N–C); 1174.50 (C–N–C); 1120.82; 1044.32; 891.32. ^1^H NMR (500 MHz, CDCl_3_): 13.51 (s, 1H, O–H), 7.92–7.90 (d, 2H, *J* = 9.0 Hz, Ar–H), 7.83-7.82 (d, 2H, *J* = 7.5, Ar–H), 7.45–7.36 (m, 5H, Ar–H), 3.60–3.52 (m, 2H, –NCH_2_^−^), 2.60–2.57 (t, 2H, *J* = 15.5, Ar–CH_2_^−^), 1.79 (m, 1H, –CH–), 1.67 (m, 2H, –CH_2_^−^), 1.23 (m, 10H, –CH_2_^−^), 0.92-0.88 (t, 3H, *J* = 15.0, –CH_3_), 0.86-0.80 (m, 6H, –CH_3_). ^13^C NMR (500 MHz, CDCl_3_): 158.65, 152.72, 149.92, 143.49, 130.93, 129.13, 124.73, 122.82, 115.80, 47.67, 38.45, 32.94, 30.62, 28.77, 26.21, 23.41, 14.12, 10.72. MS (MALDI-TOF) (DIF) M/Z (%): 516.30 (M–H)^−^. Calc. for (C28H40N4O): 516.40.

## 4. Results and Discussion

**Figure 2:**
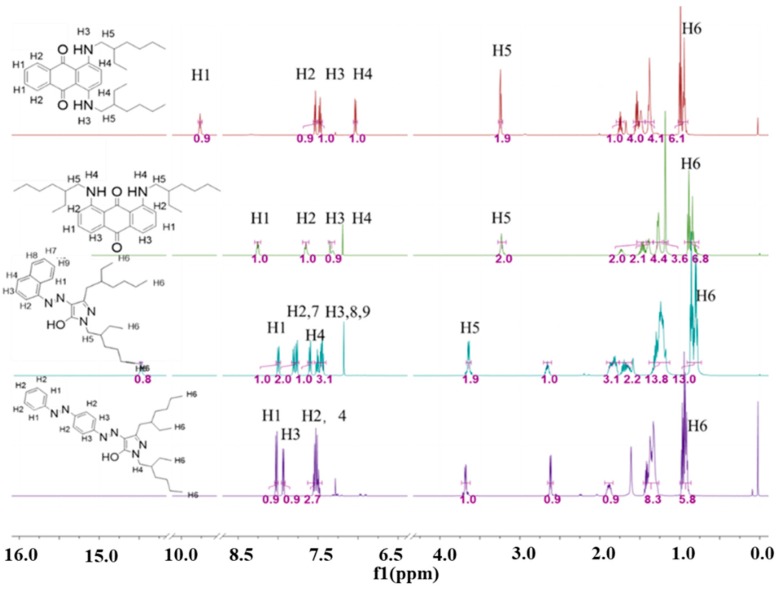
^1^H NMR spectra of four synthesized dyes.

The ^1^H NMR spectra for the target dyes recorded in CDCl_3_ are shown in Figure 2. The structures of dyes were confirmed by the presence of four distinct downfield signals characteristic of the aromatic rings. For Anthra-1–2, the structure of the anthraquinone core was confirmed by the presence of four distinct downfield signals (“H1”, “H2”, “H3”, “H4”). The “H1”, “H2”, “H3”, and “H4” protons of Anthra-1–2 exhibited one singlet, two multiplets and one doublet between 10.71 ppm and 6.93 ppm. For the substituents in the N-position, these two dyes displayed one common multiple peak “H5” between 3.22 and 3.14 ppm, corresponding to the methene protons adjacent to the imide group. The “H6” protons appeared as a singlet between 0.88 and 0.83 ppm. For the other pyrazolone dyes Pyrazolone-1–2, the “H1–4” and “H7–9” protons of Pyrazolone-1 exhibited three separate doublets, one triplet, and one multiplet between 8.00 ppm and 7.43 ppm. The “H1”, “H2”, “H3” and “H4” protons of Pyrazolone-2 exhibited three separate doublets and one multiplet between 7.92 ppm and 7.36 ppm. Two dyes: Pyrazolone-1–2, displayed one common multiple peak “H5” between 3.60 and 3.52 ppm corresponding to the methene protons adjacent to the imide group. Moreover, the common multiple peak “H6” of those two dyes represented the protons on the methyl group, and the “H6” protons existed between 0.78 and 0.92 equivalent to the twelve protons which were obtained. The chemical values confirmed the structural correctness of the four dyes.

### 4.1. Absorption Properties of Four Dyes

**Figure 3:**
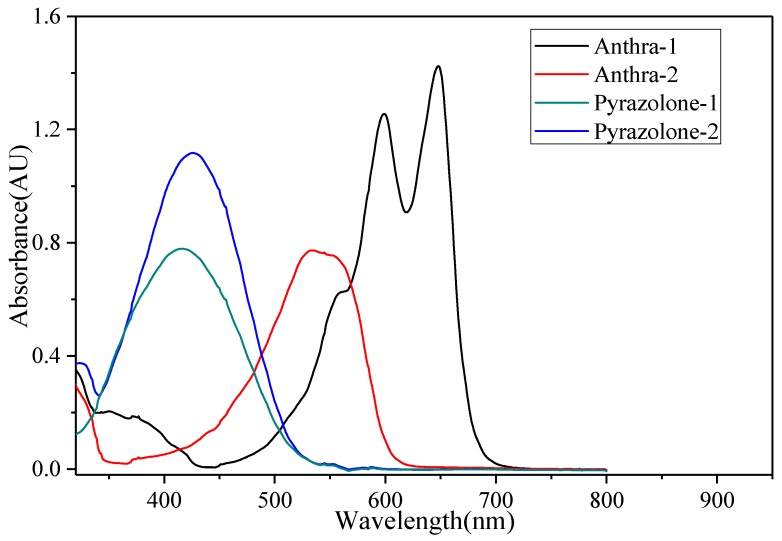
UV-visible absorption spectra of four dyes.

As can be seen in Figure 3, the uv-visible absorption spectra and data of all the dyes were recorded in decane and were found in the range of 416–650 nm, showing color variations from yellow to cyan. For the anthraquinone dyes Anthra-1–2, the maximum wavelength number (*λ*_max_) value depended on the position of the alkyl-amino substituented on the anthraquinone ring. When two alkyl-amino groups were substituted on 1,4-sites, the *λ*_max_ was 650 nm, while the *λ*_max_ was 545 nm when they were substituted on 1,8-sites. For azo pyrazolone dyes Pyrazolone-1~2, the *λ*_max_ value depends on the conjugate rings. The *λ*_max_ of Pyrazolone-1, which is a mono-azo dye with a naphene group, is 416 nm. While Pyrazolone-2 is a bisazo dye with two benzene groups, the size of conjugate rings is a bit larger than Pyrazolone-1, thus the *λ*_max_ values (420 nm) are a bit longer than for Pyrazolone-1. 

### 4.2. Photoelectric Response Properties of Dyes

**Figure 4:**
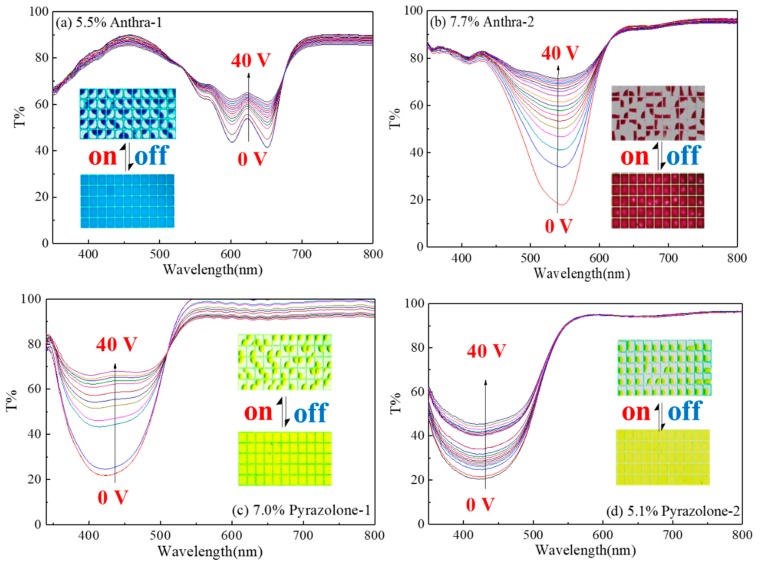
Transmission spectra of EFD display formulated by four formulated dyes under different voltages. (Insert: micron photos of pixels filled with different oils). (**a**) 5.5% Anthra-1, (**b**) 7.7% Anthra-2.

The EFD cell was fabricated using normal processes. The switching behavior of the EFD cells was studied under a certain dc voltage. The transmission spectra of EFD displays were tested under different voltages. The results can be seen in Figure 4. For Pyrazolone-1 as an example, we can clearly see the transmission changes as the voltage rose to 20 V, showing that the oil film starts to break up at this voltage. By increasing the voltage from 0 V to 40 V, the transmission value of EFD cell at maximum wavelength (420 nm) increases from 20% to 70%, showing that the cell could switch from bright yellow color (under 0 V) to the most transparent or white color (under 40 V). The EFD display could not be changed to an obvious one transparent to the naked eye until the voltage reached 32 V. And the transmission curves did not change greatly under the higher voltage because of the saturation of the oil contraction. The micron photos of EFD displays at 0 V (the OFF state) and 32 V (the ON state) are shown in Figure 4. The aperture ratio was achieved by calculating the occupying area of oil and white area in Photoshop software, and the aperture ratio could reach as high as 68.5%–75.0%. 

### 4.3. Photo-Stability Research of Dyes

The photo-stability test of EFD oils were evaluated following the standard method 60068-2-9. The aging test of the display was carried under accelerated sunlight conditions for 100 h at 45 °C, which can translate to several years of application lifetime under normal conditions [19]. 

**Figure 5:**
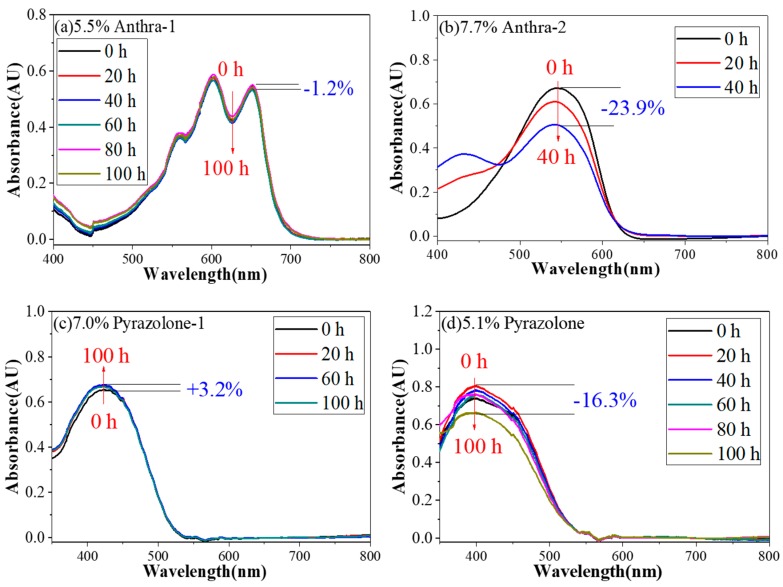
Photo-stability test results of four formulated dyes. (Irradiation condition: 0.55 W/m^2^ (340 nm); Temperature 45 °C). (**a**) 5.5% Anthra-1, (**b**) 7.7% Anthra-2, (**c**) 7.0% Pyrazolone-1, (**d**) 5.1% Pyrazolone.

The change of absorbance curves under different irradiation time is shown in Figure 5. The decrease of dye absorbance after a period of irradiation test depends on the degree of dye degradation. Generally, we can see that dye degradation of Anthra-2 and Pyrazolone-2 are higher than Anthra-1 and Pyrazolone-1. After 100h irradiation, only 1.2% of Anthra-1 dyes were decomposed. The good photo-stability of Anthra-1 was attributed to the substitution of –NH at 1,4-sites of anthrquinone structure, which could form two six-membered rings with two carbonyl groups. This special structure could strengthen the electron delocalization and decrease the electron density of potentially destabilizing groups, such as C–N and C–O bonds. In contrast, 23.9% of Anthra-2 decomposed after only 40 h of irradiation. The structure of Anthra-2 is a 1,8- sites of anthrquinone, while only one six-membered ring could be formed at the 1-site. As a result, the other substitution of –NH at the 8-site could not be stabilized and was prone to decomposing under irradiation. The initial anthraquinone dyes underwent a hydroxylation of the alkyl carbon atom adjacent to the left hand nitrogen atom. This resulted in a photo-bleached molecule with the original alkly group of the left hand alkl amine now substituted with the group –CHO [20].

For azo pyrazolone dye Pyrazolone-1–2, it could be seen that after 20 h of irradiation, the absorption values of Pyrazolone-1 and Pyrazolone-2 increased by 3.2% and 10%, respectively. This abnormal phenomenon could be explained by the transformation of cis-tran isomerization of azo group caused by light. However, when the irradiation time was extended to 100 h, the absorption increase ratio R% of Pyrazolone-1 was maintained at around 3.2%, while the absorption of Pyrazolone-2 decreased to 83.7%, showing that 16.3% of Pyrazolone-2 was decomposed and few Pyrazolone-1 molecules were decomposed. This difference could also be explained by the difference of structures. Pyrazolone-2 is a bisazo dye, and the azo group adjacent to the pyrrazole structure could form a six-membered ring, while the other isolation azo group could be easily destroyed by strong photo irradiation. Pyrazolone-1 is a monoazo dye, meaning the azo group adjacent to pyrrazole structure could form a six-membered ring and was stabilized. Thus, it is much more stable than Pyrazolone-2.

**Figure 6:**
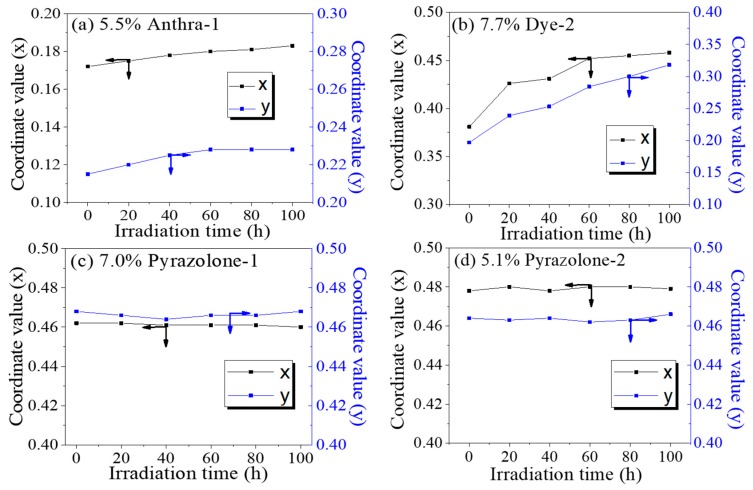
Variation of color coordinates (x, y) of oils in CIE1931 color space and *ΔE*. (Irradiation condition: 0.55 W/m^2^ (340 nm); Temperature 45 °C). (**a**) 5.5% Anthra-1, (**b**) 7.7% Dye-2, (**c**) 7.0% Pyrazolone-1, (**d**) 5.1% Pyrazolone-2.

The color coordinates (x, y) of colored EFD cell in CIE1931 color space and color deviation *ΔE* were monitored along with the accelerating irradiation test. The results could be seen in Figure 6 and Table 1. With the exposure of irradiation time, the color coordinates (x, y) of the colored EFD cell in the CIE1931 color space changed, showing changes of the color. Among the four colored EFD oils, the color coordinates (x, y) of Anthra-2 had the greatest change from (0.38, 0.20) to (0.45, 0.32), showing that the color changed from bright magenta to brown. The *ΔE* of Anthra-2 was found to be as high as 130.5 after 100 h of irradiation. The color coordinates (x, y) of Pyrazolone-1 changed from (0.462, 0.468) to (0.460, 0.468), showing that the color did not change anymore. The *ΔE* of Pyrazolone-1 was 2.0, showing a small color change which can hardly be distinguished by the naked eye. The color coordinates (x, y) of Anthra-1 and Pyrazolone-2 changed from (0.172, 0.215), (0.478, 0.464) to (0.183, 0.228), (0.479, 0.466), respectively. The *ΔE* of Anthra-1 and Pyrazolone-2 were 7.7 and 3.7, respectively, showing a slight color change. These performances are highly relevant to their structure stability. Structure of Anthra-2 is the least stable and its color changed a great deal, while structure of Pyrazolone-1 is the most stable and its color changed minimally. 

### 4.4. Effect of Irradiation on Backflow Property of EFD Devices

The tendency of the oil to flow back into the pixel at a constant voltage is called “backflow”. The reduction oil-level contraction affects pixel aperture for the backflow of oil, which has an impact on the ability of the switch and the power consumption of the display. Since a static reading is the mode of the operation for an EFD display, a low “backflow” property oil will lead to the decrease of the refresh rate for the display. Thus, the power consumption can be reduced because of its scaling with the refresh rate. 

**Figure 7:**
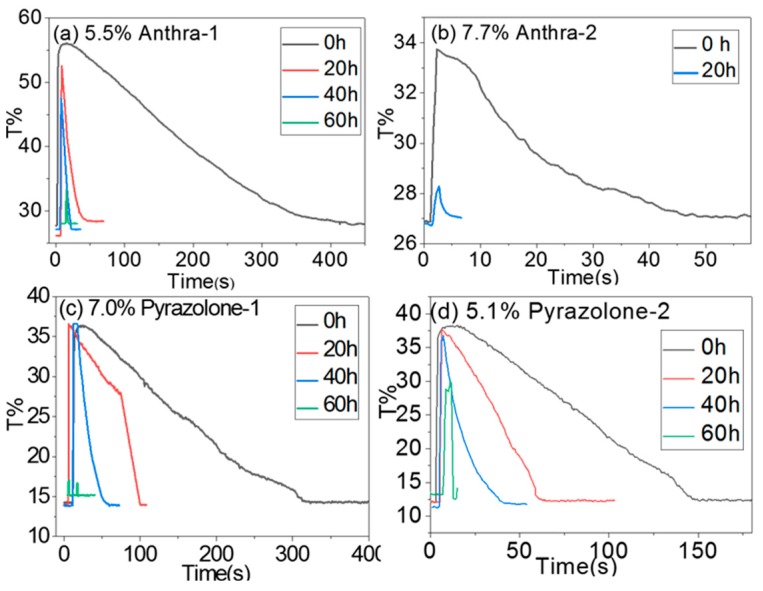
Backflow properties of EFD devices with dyes under different irradiation times. (Irradiation condition: 0.55 W/m^2^ (340 nm); Temperature 45 °C). (**a**) 5.5% Anthra-1, (**b**) 7.7% Anthra-2, (**c**) 7.0% Pyrazolone-1, (**d**) 5.1% Pyrazolone-2.

In Figure 7, it can be seen that before irradiation, the backflow time of four oils were around 50–400 s under 30 V DC (Direct Current) voltage, which is rather long and acceptable. The faster refresh speed for such a low backflow property will lead to a static image in an EFD display. A long backflow time can be attributed to the non-polarity of dye structures. The discrimination of backflow time for four oils is due to the difference of the non-polarity of their structures. When EFD cells underwent irradiation for a certain period, the backflow time of four dyes deteriorated sharply. For Anthra-2, the cell cannot be open after only 20 h of irradiation. This result might cause a dye’s decomposition. When Anthra-2 was decomposed under irradiation, small fragments were released into oil, resulting in the rise of oil conductivity, thus the backflow time was extremely short and even pixels could not be opened. For Anthra-1 and Pyrazolone-1, in spite of the least dye decomposition, the backflow time also decreased from ~400 s and ~300 s to ~15 s and ~100 s, respectively after only 20 h of irradiation, and cells could not opened after 60 h of irradiation. These results showed that the effect of irradiation on the backflow property of EFD devices is not only relevant to a dye’s structure, but also relates to the other materials, such as pixel wall materials and fluoropolymer materials. Any decomposition of these materials will release small high polarity fragments into oil, causing a rise in oil conductivity, and a decrease of the backflow time of EFD cell. This effect will be studied in-depth by our group, so it will not be extensively discussed in this paper. 

## 5. Conclusion

In summary, we have synthesized four EFD oil materials based on anthraquinone and azo pyrazolone dyes. The absorption, electrical-optical response, and photo-stability of these dyes were researched in detail. The dyes have good electrical-optical response properties due to their low polarity of whole molecules. However, the photo-stability was highly dependent on the structures. High polarity and high electronic density of the chemical bond are the photo-sensitive groups which are potentially destroyed under photo irradiation. Six-membered rings formed by hydrogen bonds could play an important role as a “protective structure”, and they can highly increase the photo-stability property of these dyes. Irradiation could sharply decrease the backflow time of existing EFD devices, which is not only caused by the decomposition of oil materials, but also may be caused by the other materials in the pixels.

## Figures and Tables

**Table 1:** Color change (*ΔE*) of four formulated dyes after accelerated irradiation.

Dye	Before Irradiation			After Irradiation			*ΔE*
	*L*	*a*	*b*	*L*	*a*	*b*	
Anthra-1	174.9	−45.5	−123.6	171.2	−43.5	−117.1	7.7
Anthra-2	140.8	181.0	−68.2	160.7	113.2	41.6	130.5
Pyrazolone-1	236.2	14.0	210.5	236.3	12.3	209.3	2.0
Pyrazolone-2	233.3	29.7	221.4	231.9	28.1	224.6	3.7

(Irradiation condition: 0.55 W/m^2^ (340 nm); Irradiation time: 100 h; Temperature 45 °C).
